# Challenges in Toxocariasis Diagnosis: From Pericarditis, through Hepatic Tumor, to the Detection of Brain Aneurysms: Case Report

**DOI:** 10.3390/pathogens13030254

**Published:** 2024-03-15

**Authors:** Martyna Biała, Joanna Nieleńczuk, Anna Chodorowska, Bartosz Szetela

**Affiliations:** 1 Department of Infectious Diseases, Liver Diseases and Acquired Immune Deficiences, Wroclaw Medical University, 51-149 Wroclaw, Poland; 2 Affidea Medical Diagnostic Imaging Centre, 53-439 Wroclaw, Poland

**Keywords:** toxocariasis, neurotoxocariasis, aneurysms, parasitosis

## Abstract

Toxocariasis is the parasitic infection caused by the larvae of *Toxocara* roundworms species: *Toxocara canis* from dogs and, less frequently, *Toxocara cati* from cats. The high proportion of asymptomatic cases of toxocariasis and the uncharacteristic clinical manifestations mimicking other medical conditions make diagnosis challenging. The main clinical presentations of toxocariasis are visceral and ocular larva migrans. Migration to the central nervous system (neurotoxocariasis) is rare and can cause meningitis, encephalitis, myelitis, cerebral vasculitis, seizures, headache or asymptomatic CNS infection. Neurotoxocariasis is an uncommon diagnosis and it is probably underdiagnosed due to the nonspecific clinical manifestations, low awareness of physicians as well as the lack of standardized diagnostic exams. To date, no causality has been proven between neurotoxocariasis and aneurysms, but due to the character of immune response elicited by the parasites, it remains an important possibility for further research. We present a case report of a woman infected with *Toxocara canis* highlighting the diagnostic difficulties. We aim to raise the awareness of the clinical symptomatology of neurotoxocariasis.

## 1. Introduction

Toxocariasis is the parasitic infection caused by the larvae of *Toxocara* roundworms species: *Toxocara canis* from dogs and, less frequently, *Toxocara cati* from cats [1]. Humans become accidental hosts after ingesting infective eggs either directly or by eating undercooked meat of infected parasitic hosts, such as rabbit, chicken, swine or cattle [1]. Toxocariasis is more common in developing countries, mainly in tropical regions [2]. Seroprevalence of *Toxocara* spp. in the European region was estimated at 10.5% [2]. The high proportion of asymptomatic cases of toxocariasis and the uncharacteristic clinical manifestations that mimic different medical conditions make diagnostic workup challenging. Here, we present a 58-year-old patient who developed toxocariasis accompanied by brain aneurysms.

## 2. Materials and Methods

We conducted a retrospective descriptive study of medical record documentation of the 58-year-old patient. All data analyzed were collected as a part of routine diagnosis and treatment in an outpatient clinic and our analysis looked retrospectively at outcomes. Verbal informed consent was obtained prior to the study. The patient agreed to share anonymous data of her test results.

## 3. Description of Case

**Figure 1:**
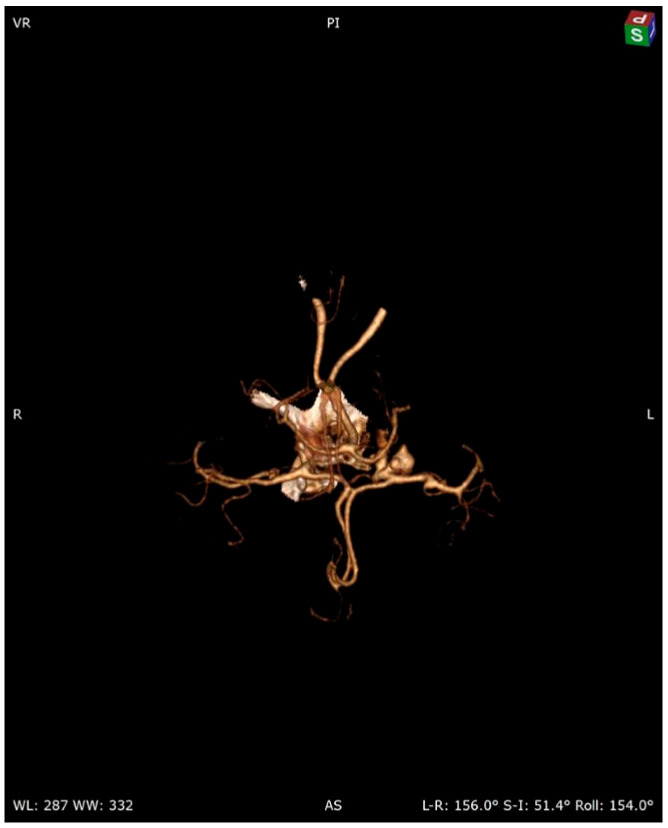
Cerebral 3D CT angiography.

**Figure 2:**
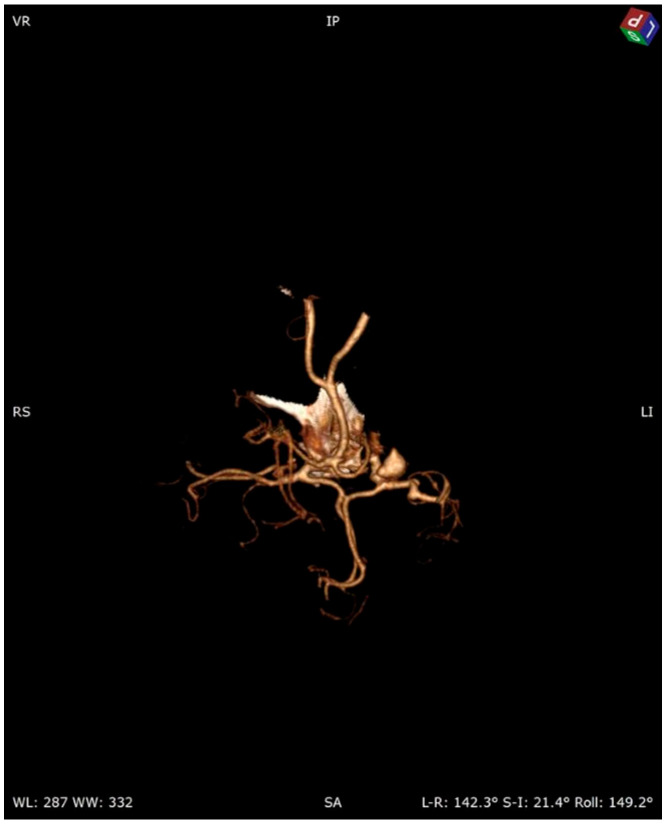
Cerebral 3D CT angiography.

**Figure 3:**
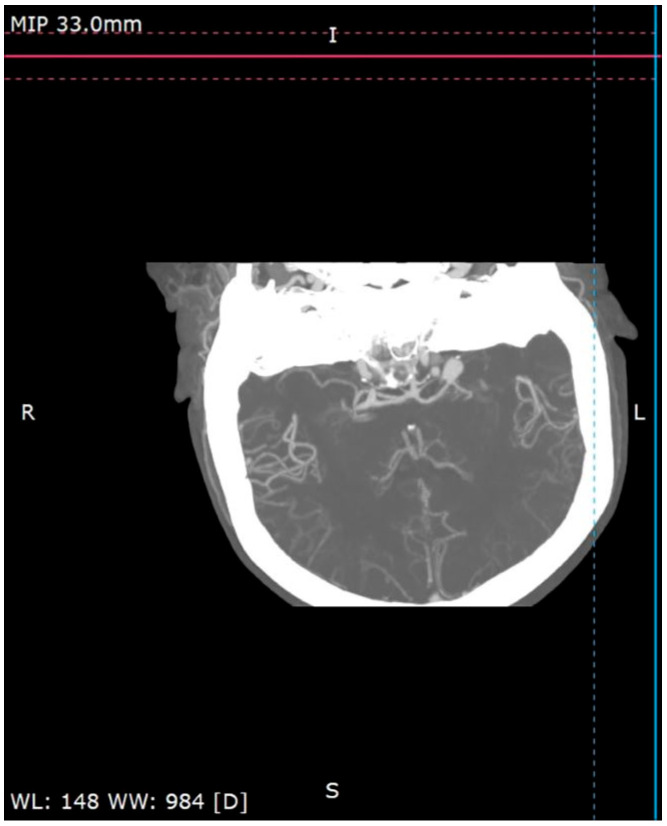
Cerebral CT angiography.

A 58-year-old woman treated for exudative pericarditis (effusion up to 6 mm) for the last 6 months was referred to a hepatologist/infectious diseases specialist with hypoechoic unclear lesions in the liver due to suspected hepatic tumor. Computed tomography confirmed simple hepatic cysts, while blood analysis revealed significant eosinophilia (23.4% in percentage, normal range 1–6%; eosinophil count: 1.45 in thousand eosinophils per microliter (K/μL), normal range 0.02–0.5 K/μL). The patient denied any symptoms, such as fever, abdominal pain, weight loss, cough or diarrhea in recent months; however, she described left-sided subjective eyeball enlargement, pain and redness. Her history included travels to Mexico, Egypt and Tunisia, as well as occasional raw meat consumption in Poland. She also had a dog, which did not receive regular parasite screenings and preventive medications. Her father had died from liver cancer. Lab tests confirmed the presence of IgG antibodies against *Toxocara canis* and sustained eosinophilia (18.1% in percentage, normal range 1–6%; eosinophil count: 1.01 in thousand eosinophils per microliter (K/μL), normal range 0.04–0.4 K/μL). Echinococcosis was excluded based on negative serology. Diagnosis of toxocariasis was further supported by clinical manifestations and exposure history with positive serology, and the patient was treated with mebendazole (200 mg twice a day for 5 days). Due to the suspicion of an ocular parasitic invasion, she was sent for an ophthalmological consultation. The ophthalmological examination was inconclusive, but head MRI revealed vascular abnormalities in brain arteries. CT angiography confirmed four aneurysms with one causing the constriction of an artery supplying the left eyeball (Figure 1, Figure 2 and Figure 3): two cerebral aneurysms of LICA (left internal carotid artery) size: 4.2 × 8.7 × 3.5 mm and 9.8 × 7.8 × 10.7 mm, one cerebral aneurysm of LMCA (left middle cerebral artery) size: 3.5 × 3.3 × 3.2 mm and the fourth enlargement for differentiation between an aneurysm and an infundibular dilations (3 mm) of LMCA. Clipping the aneurysms dissolved the ophthalmological problems. During the last check-up, normalization of the eosinophil count, full resolution of the ocular symptoms and no pericardial effusion were seen, further supporting our diagnosis of visceral toxocariasis. Hepatic cysts began to disappear. The patient was recommended to perform an abdominal ultrasound every 6 months.

## 4. Discussion

Several factors that have been associated with the risk of toxocariasis include eating raw or undercooked meat, raw vegetables or fruits that have been contaminated with wastewater or grown in contaminated soil, or owning pets (dogs, cats). Data indicate that children and young people are at a higher risk of toxocariasis than adults [3]. This may be a result of poor hand hygiene, geophagia and fun and games in outdoor environments, such as sandboxes and other playgrounds where dog and cat feces can be found. In our case, the diagnosis of toxocariasis was based on clinical manifestations, exposure history (raw meat consumption, owning a dog with no regular parasite screening, travel to increased risk countries), positive serology and eosinophilia. Clinical manifestations of toxocariasis range from asymptomatic infection to multiple organ injury. Although *Toxocara* roundworms species are known to cross the blood-brain barrier, central nervous system (CNS) involvement is uncommon. Nevertheless, the hypothesis that asymptomatic cerebral toxocariasis may progress to neurodegenerative diseases such as Alzheimer’s disease cannot be ignored [4]. CNS manifestations include meningitis, encephalitis, myelitis, cerebral vasculitis, seizures, headache or asymptomatic CNS infection. The neurological problems caused by *Toxocara* spp. are noted mostly in low-income countries; however, sporadic cases can also be detected in non-endemic regions. Many early symptoms of neurotoxocariasis are often nonspecific and the total number of *Toxocara* cases might be underdiagnosed. According to Dzikowiec, M. et al., the most common symptoms reported by patients with parasitic CNS invasions include headache, dizziness, epileptic seizures, sensory disturbances, increased intracranial pressure, meningeal syndrome and cerebellar ataxia [5]. Studies have also found an association between positive *Toxocara* spp. serology and epilepsy, neurodegeneration, cognitive delay or psychiatric illness [6,7,8,9]. Moreover, Hill et al. presented a case of a 2.5-year-old British infant with a bilateral subdural hematoma and subarachnoid hemorrhage, and the autopsy revealed *Toxocara canis* larvae in the brain [10]. Sandal et al. reported a case of *Toxocara* infection in an 8-year-old child who presented with intermittent fever, hypereosinophilia complicated by massive pericardial effusion and a mycotic aneurysm, and the possibility of the mycotic aneurysm due to neurotoxocariasis was considered [11]. To date, no causality has been proven between neurotoxocariasis and CNS aneurysms, but due to the character of immune responses elicited by the parasites, it remains an important possibility for further research. Diagnosis of neurotoxocariasis is mainly based on the detection of specific antibodies against *Toxocara* spp. in CSF or serum, and clinical and radiological improvement after antiparasitic therapy. However, definitive diagnosis is based only on histological confirmation which is rarely available [7]. Treatment is based on antiparasitic drugs, commonly albendazole or mebendazole. In our case, clipping the aneurysms dissolved the ophthalmological problems. After antiparasitic therapy with mebendazole, the normalization of the eosinophil count and full resolution of pericardial effusion were seen. Hepatic cysts also began to disappear. Cerebrospinal fluid collection by lumbar puncture is performed in the diagnostic workup of neurotoxocariasis, but our patient did not undergo this procedure, and that factor limits the study.

## 5. Limitations

Diagnosis of this patient was particularly challenging due to the presence of a wide range of symptoms. We thought the risk of diagnostic pericardial fluid, CSF or ocular sampling, as well as liver biopsy for molecular or histology exams were too high given the patient’s good general condition, despite the long-standing history, and quick improvement with no recurrence after dedicated treatment. However, a lack of these tests might limit our diagnosis, analysis and conclusions.

## 6. Conclusions

This case should prompt more attention to neglected parasitic diseases and the necessity to include them in differential diagnoses. Toxocariasis should be considered in any patient presenting with persistent hypereosinophilia with multisystem involvement. However, only the detailed examination and holistic approach can lead to correct diagnosis and treatment. The possibility of aneurysm development due to chronic immune-inflammatory states elicited by parasitic infections needs further evaluation.

## Data Availability

The data presented in this study are available and can be shared on reasonable request sent to the corresponding author.
